# PNO1 inhibits autophagy-mediated ferroptosis by GSH metabolic reprogramming in hepatocellular carcinoma

**DOI:** 10.1038/s41419-022-05448-7

**Published:** 2022-11-29

**Authors:** Xiaomeng Hu, Yuchao He, Zhiqiang Han, Wei Liu, Dongming Liu, Xihao Zhang, Lu Chen, Lisha Qi, Liwei Chen, Yi Luo, Qiang Li, Peng Chen, Qiang Wu, Xiaolin Zhu, Hua Guo

**Affiliations:** 1 Department of Tumor Cell Biology, Tianjin Medical University Cancer Institute and Hospital, National Clinical Research Center for Cancer, Key Laboratory of Cancer Prevention and Therapy, Tianjin’s Clinical Research Center for Cancer, 300060 Tianjin, China; 2 Department of Hepatobiliary Cancer, Liver Cancer Research Center, Tianjin Medical University Cancer Institute and Hospital, National Clinical Research Center for Cancer, Key Laboratory of Cancer Prevention and Therapy, Tianjin’s Clinical Research Center for Cancer, 300060 Tianjin, China; 3 National Clinical Research Center for Cancer, Key Laboratory of Cancer Prevention and Therapy, Tianjin’s Clinical Research Center for Cancer, 300060 Tianjin, China; 4 Department of Anesthesiology, Tianjin Medical University Cancer Institute and Hospital, National Clinical Research Center for Cancer, Key Laboratory of Cancer Prevention and Therapy, Tianjin, Tianjin’s Clinical Research Center for Cancer, Key Laboratory of Cancer Immunology and Biotherapy, 300060 Tianjin, China; 5 Department of Pathology, Tianjin Medical University Cancer Institute and Hospital, National Clinical Research Center for Cancer, Key Laboratory of Cancer Prevention and Therapy, Tianjin’s Clinical Research Center for Cancer, 300060 Tianjin, China; 6 Department of Thoracic Oncology, Lung Cancer Diagnosis and Treatment Center, National Clinical Research Center for Cancer, Key Laboratory of Cancer Prevention and Therapy, Tianjin’s Clinical Research Center for Cancer, 300060 Tianjin, China

**Keywords:** Oncogenes, Cell death

## Abstract

Effective strategies for hepatocellular carcinoma, which is the second leading cause of death worldwide, remain limited. A growing body of emerging evidence suggests that ferroptosis activation is a novel promising approach for the treatment of this malignancy. Nevertheless, the potential therapeutic targets and molecular mechanisms of ferroptosis remain elusive. In this study, we found that PNO1 is a bona fide inhibitor of ferroptosis and that autophagy induced by PNO1 promotes cystine/glutamate antiporter SLC7A11 while increasing the synthesis and accumulation of intracellular glutamate. This increase is followed by an equally proportional addition in cystine uptake, which consequently enhances system Xc^-^ activity that leads to the inhibition of ferroptosis. In the maintenance of redox homeostasis, system Xc^-^ activated via PNO1-autophagy metabolism is responsible for maintaining cysteine for glutathione (GSH) synthesis, and the final GSH metabolic reprogramming protects HCC cells from ferroptosis. The combination of PNO1 inhibition with drugs causing ferroptosis induction, particularly sorafenib, the first-line drug associated with ferroptosis in liver cancer shows therapeutic promise in vitro and in vivo. Together, our findings indicated that PNO1 protects HCC cells from ferroptotic death through autophagy-mediated GSH metabolic remodeling, and we identified a candidate therapeutic target that may potentiate the effect of ferroptosis-based antitumor therapy.

## Introduction

Hepatocellular carcinoma (HCC) is the second cause of malignant cancer death, particularly in China [1]. It has high incidence and mortality rates and lacks efficient therapy [2]. Indeed, given that the liver is a hub for glucose, lipid, and amino acid metabolism [3], the metabolic dysfunction and ROS inhibition that are triggered to maintain redox homeostasis protect tumor cells from death when they are exposed to conventional chemotherapy and radiation [4]. Notably, an increasing number of reports have suggested a novel therapeutic opportunity that targets ferroptosis, a new form of cell death that is regulated by cellular metabolism, redox homeostasis, and signaling pathways related to cancer [5–7]. Although the physiological function of ferroptosis remains obscure, numerous studies have shown that the activation of ferroptosis contributes to various cancer treatments, such as immune checkpoint blockade [8] and radiotherapy [9], indicating that ferroptosis plays an important role in tumor suppression. Furthermore, traditional drug-resistant tumor cells are still vulnerable to ferroptosis inhibitors [10, 11]. However, sorafenib, the ferroptosis-linked clinical drug applied in HCC despite its limited benefits and common resistance, is not a bona fide inducer of ferroptosis because it cannot induce widespread ferroptosis in all HCC cell lines [12]. Given the above-mentioned facts, discovering new ferroptosis target genes and their underlying mechanism in HCC is urgently required.

Studies on identifying the molecular regulators, in addition to cystine/glutamate metabolic transporters (SLC7A11 and xCT) and the final key peroxidase GPX4 involved in the synthesis of GSH, that regulate ferroptosis have increased [13]. Autophagy meets the altered metabolism and energy requirements of tumors by regulating the digestion of intracellular macromolecules and organelles [14]. Furthermore, ample support for the claim that the results of metabolic reprogramming and the regulation of redox requirements generated by autophagy may affect ferroptosis exists. However, the exact mechanism that regulates ferroptosis and network cascades between autophagy and ferroptosis is unclear.

In the present study, we observed that in HCC cell lines, the RNA-binding protein partner of NOB1 (PNO1) plays a fundamental role in GSH metabolic reprogramming against ferroptosis by promoting autophagy. Mechanistically, PNO1 increases intracellular glutamate by promoting autophagy, which utilizing macromolecules and organelles to resynthesize new amino acids. The increment in intracellular glutamate could activate system Xc^-^. Then, the uptake of cystine is increased, and the biosynthesis of GSH expands. Further analysis showed that the suppression of PNO1 increases the sensitivity of tumor cells to ferroptosis by inhibiting autophagy. We also demonstrated that PNO1 inhibition repressed SLC7A11 transcription through p53 to promote ferroptosis. In conclusion, this research critically evaluates PNO1 as a negative modulator of ferroptosis and provides a potential therapeutic strategy that targets PNO1 to strengthen sorafenib sensitivity against ferroptosis in HCC.

## Material and methods

### Antibodies and reagents

The following antibodies were used: anti-PNO1 was from Santa Cruz Biotechnology, anti-β-actin was from Cell Signaling Technology, anti-SLC7A11/Xc^-^ from Proteintech, anti-GPX4 from Bioss antibodies; anti-LC3B, anti-SQSTM1/p62 from Cell Signaling Technology (Beverly, Massachusetts). Erastin (S7242), ferrostatin-1 (S7243), and RSL3(S8155) were all purchased from Selleck Chemicals. P62Sorafenib(S125098) was obtained from Aladdin. BODIPY 581/591(D3861) was from Invitrogen; DCFH-DA was from Beyotime; DMEM was from Corning.

### Cell transfection

The packaging plasmids and expression plasmids (sh-PNO1#1, sh-PNO1#2, sh-Ctrl, sh-p53 and sh-Ctrl, PNO1 and Vector) were transfected into HEK293T cells. Then Hep3B and HLE cells were infected with a lentivirus to produce stable PNO1 KD or OE cells.

### Western blotting analysis

The cells were collected and lysed on ice with 1×SDS lysis buffer supplemented with 1 mM NaF, 1 mM Na_3_VO_4_, 1×protease, and phosphatase inhibitor cocktail (Hoffman-la Roche Ltd, Basel, Switzerland) for 30 min. The proteins were then loaded on gels and separated by SDS-PAGE. Then, proteins were transferred to PVDF membranes. After blocking with 5% non-fat milk, the membrane was incubated with various primary antibodies overnight at 4 °C, followed by incubation with secondary antibodies. The primary antibodies were: anti-PNO1(1:1000), anti-β-actin (1:1000), anti-SLC7A11/Xc^-^ (1:1000), anti-GPX4 (1:1000).

### Colony formation assay

1000 cells were plated at 12-well plates in DMEM medium supplemented with 10% FBS for the colony formation assay. After 2 weeks of incubation, the surviving colonies were fixed and stained with 0.5% crystal violet, imaged and counted.

### Cell viability assay

Cells were plated in a 96-well plate at 2000 cells/well for 24 h. Then treated with erastin or sorafenib with or without ferrostatin-1 at indicated concentrations for 24 h. Treated cells of erastin with rapamycin or chloroquine for 24 h and then added 10 μL of CCK-8 reagent (Dojindo) in each well in a 37 °C incubator for 3 h. The OD value of the wavelength at 450 nm was measured.

### Quantitative real-time polymerase chain reaction

Total RNA was isolated using TRIzol (Ambion) according to the manufacturer’s instructions. cDNA quantitative RT-PCR kit (Takara, Japan) was performed for reverse transcription. SYBR Green-based real-time PCR was using SYBR Green master mix (Takara, Japan). For analysis, the cycle threshold (Ct) values were normalized to expression levels of β-actin.

### PI staining

Cells were plated in a 12-well plate for 24 h. And then some cells treated with erastin, sorafenib with or without ferrostatin-1. Some cells treated with erastin and co-treated with rapamycin or chloroquine at indicated concentrations for 24 h. Afterward, 1 ml DMEM medium added with 1 µL of PI reagents(1 mg/ml) and Hoechst reagents for 20 min. Subsequently, cells were visualized using a fluorescent microscope.

### Immunohistochemistry

The tumor tissue microarray assay (TMA) was infiltrated in xylene, rehydrated through an ethanol series, and then retrieved the antigen in citrate. The percentage immunoreactivity score was evaluated on a 4-point scale: 0.0<10% positive cells; 1.10–40% positive cells; 2.40–70% positive cells; and 3.70–100% positive cells. The primary antibodies were anti-PNO1, anti-SLC7A11/Xc^-^.

### Lipid ROS analysis

The treatment of cells was just as same as the analysis of ROS. After centrifugation and washing, cells were resuspended in 500 μL phosphate-buffered saline (PBS) containing 5 μM C11-BODIPY (581/591) (D3861, Invitrogen), and incubated for 30 min at 37 °C in an incubator. And analyzed using a flow cytometer (FAC Suite, BD Biosciences) equipped with a 488 nm laser for excitation. The data analysis was performed by using Flow Jo software.

### Glutathione assay

The cell lysate’s relative GSH concentration was measured using an assay kit from Nanjing Jiancheng (#A006-2) according to the manufacturer’s instructions. The luminescence of yellow product of the reaction was measured at 405 nm and normalized by protein concentration.

### Glutamate assay

The intracellular glutamate concentration was measured by assay kit from Nanjing Jiancheng (#A074-1) according to the manufacturer’s instructions, and the reaction product was measured spectrophotometrically at 340 nm. The values were also normalized by protein concentration.

### Cystine uptake

The cystine uptake level was measured by assay kit from DOJINDO Laboratories (UP05-DOJINDO) according to the manufacturer’s instructions, data were normalized by CCK8 based cell number quantification.

### Cysteine assay

The intracellular and mouse tissue cysteine concentration was measured by an assay kit from Nanjing Jiancheng (#A126-1) and the product was measured spectrophotometrically at 600 nm according to the manufacturer’s instructions.

### Animal models

Xenograft mouse model experiments were used male BALB/c nude mice (4 weeks old) purchased from SPF Biotechnology (Beijing, China). Each mouse was injected 5 × 10^6^ tumor cells at the volume of 100 μL into the subcutaneous tissue. The tumor volume and weight of the mice was observed every 2 days. Mice were monitored daily and the tumor volume calculated according to the equation volume = length × width^2^ × 1/2.

### Gene set enrichment analysis

GSEA was performed to determine whether the expression of PNO1 is related to p53 pathway on the basis of GSE88402.

## Results

### PNO1 is a bona fide redox-responsive repressor of ferroptosis

**Fig. 1:**
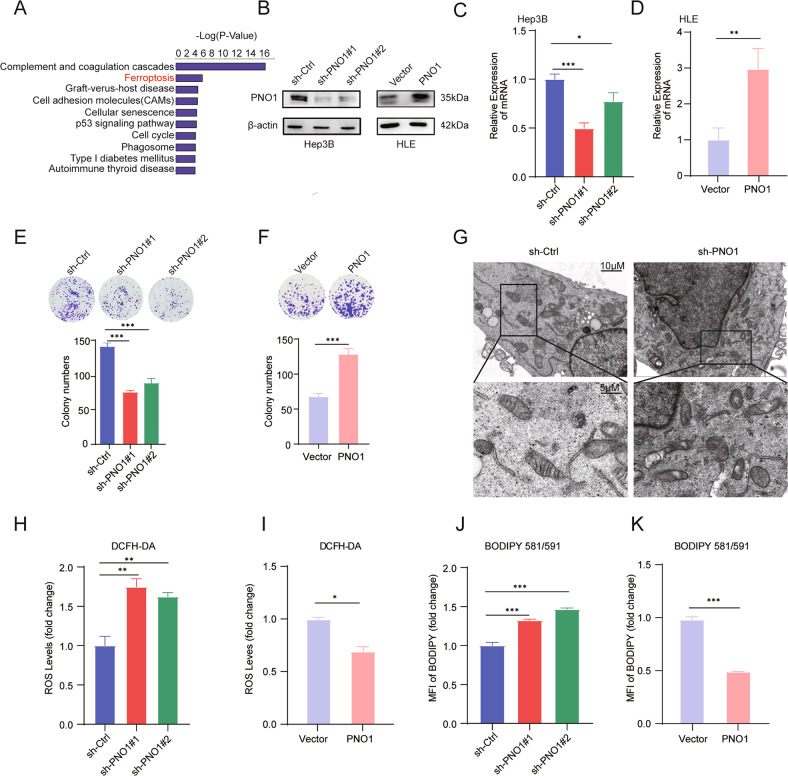
PNO1 is a negative regulator of ferroptosis in HCC. **A** The proteomic analysis of Hep3B sh-Ctrl and sh-PNO1 cells. **B** Western Blotting analysis of PNO1 protein level in Hep3B sh-PNO1 and HLE PNO1 cells compared with their parental cells. **C**, **D** The mRNA expression of PNO1 in Hep3B sh-PNO1 (**C**) and HLE PNO1 (**D**) cells was detected with qRT-PCR (*P < 0.05, **P < 0.01). **E**, **F** The colony formation assay shown the growth effect of PNO1 in Hep3B sh-PNO1 (**E**) and HLE PNO1 cells (**F**) compared with their parental cells (***P < 0.001). **G** Representative transmission electron microscopy images of Hep3B sh-Ctrl and sh-PNO1 cells. **H**, **I** The ROS levels in indicated Hep3B (**H**) and HLE cells were assayed by DCFH-DA staining (**I**) (**P < 0.01, *P < 0.05). **J**, **K** The lipid ROS levels in indicated Hep3B (**J**) and HLE cells were measured by BODY staining (**K**) (***P < 0.001).

Our previous study has shown that PNO1 is associated with the progression of various cancers, including HCC [15] and lung adenocarcinoma [16]. We further explored the molecular mechanism of PNO1 in regulating the malignant progression of HCC by performing differential protein enrichment analysis, which suggested that PNO1 was significantly associated with ferroptosis (Fig. 1A). Additionally, Hep3B and HLE cells were used to establish stable PNO1 downregulation (sh-PNO1) and PNO1 upregulation (PNO1) cell lines and their control cells sh-Ctrl and Vector, respectively, to verify whether PNO1 can regulate ferroptosis. Western Blotting (WB) analysis and quantitative real-time polymerase chain reaction (qRT-PCR) were used to confirm the efficiency of PNO1 deletion and overexpression (Fig. 1B–D). Then, colony formation assay was used to assess the effect of PNO1 on HCC cell viability. Our results showed that relative to the cell viability of control group cells, that of Hep3B sh-PNO1 cells decreased, whereas that of HLE PNO1 cells increased (Fig. 1E, F). These results demonstrated that PNO1 promoted cell viability of HCC cell lines. Ferroptosis is characterized by the morphological features of shrunken mitochondria with increased membrane density [17]. We observed the changes in Hep3B sh-PNO1 cell lines through transmission electronic microscopy (Fig. 1G). We measured cellular ROS and lipid ROS levels via flow cytometry to investigate the effect of PNO1 on ferroptosis. DCFH-DA staining revealed that the suppression of PNO1 expression significantly promoted the accumulation of ROS. Conversely, PNO1 overexpression decreased ROS levels in HLE cell lines (Fig. 1H, I). Similar results were observed when we quantified the levels of lipid ROS via C11-BODIPY staining (Fig. 1J, K). All these results suggested that PNO1 itself may operate as a redox-responsive suppressor and that its inhibition can promote ferroptosis to limit tumor growth in HCC cell lines.

**Fig. 2:**
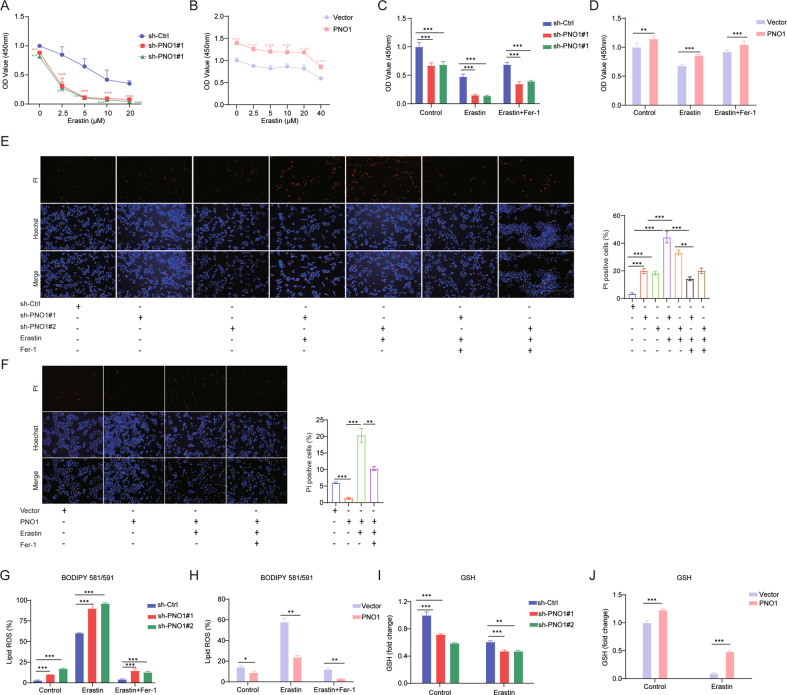
PNO1 suppression enhances ferroptosis in HCC cell lines. **A**, **B** Cell viability was assayed in Hep3B sh-PNO1 cells (**A**) and HLE cells (**B**) treated with or without erastin (0–20 μM for Hep3B, 0–40 μM for HLE) for 24 h controlled with their parental cells (**P < 0.01, ***P < 0.001). **C**, **D** Cell viability was assayed in indicated Hep3B (**C**) and HLE (**D**) cells treated with or without erastin (5 μM for Hep3B, 10 μM for HLE) and ferrostatin-1 (5 μM) for 24 h (***P < 0.001, **P < 0.01). **E**, **F** Cell death was assayed in indicated Hep3B (**E**) and HLE (**F**) cells treated with or without erastin (5 μM for Hep3B, 10 μM for HLE) and ferrostatin-1 (5 μM) for 24 h by PI staining (***P < 0.001, **P < 0.01). **G**, **H** The lipid ROS levels were assayed in indicated Hep3B (**G**) and HLE (**H**) cells treated with or without erastin (5 μM for Hep3B, 10 μM for HLE) and ferrostatin-1 (5 μM) for 24 h (***P < 0.001, *P < 0.05, **P < 0.01). **I**, **J** The relative GSH levels were measured in indicated Hep3B (**I**) and HLE (**J**) cells treated with or without erastin (5 μM for Hep3B, 10 μM for HLE) for 24 h (***P < 0.001, **P < 0.01).

Erastin, a small-molecule ferroptosis inducer, has been proven to have a pivotal role in inducing ferroptosis [18, 19]. We treated HCC cell lines with different concentrations of erastin and validated that sh-PNO1 enhanced erastin-induced ferroptotic cell death. As expected, we found that the suppression of PNO1 expression markedly decreased the viability of cancer cells and cell viability in PNO1-overexpressing cells increased relative to that in their parental cells after erastin treatment (Fig. 2A, B). Similar results could be observed by using RSL3, another iron death inducer [20] (Supplementary Fig. 2A, B), suggesting that sh-PNO1 enhanced ferroptosis-induced cell death. Notably, in the presence of erastin, PNO1 suppression increased erastin-induced cell death and lipid ROS accumulation, whereas these effects could be rescued by treatment with the ferroptosis inhibitor ferrostatin-1 (Fer-1) (Fig. 2C, E, G). However, the opposite results were observed in PNO1 overexpression cells (Fig. 2D, F, H). Therefore, under exposure to ferroptosis stimulation, sh-PNO1 dramatically amplified ferroptosis induction.

Given that ferroptosis is caused by the loss of cell redox balance, glutathione (GSH) plays an important role in eliminating the accumulation of lipid ROS [20, 21]. We next investigated whether PNO1 KD is related to GSH synthesis and quantified the levels of GSH with or without erastin treatment. Our results suggested that GSH levels were suppressed in Hep3B sh-PNO1 cells and promoted in HLE PNO1 cells (Fig. 2I, J). Moreover, similar results were found for RSL3-induced cell death (Supplementary Fig. 1C, D) and lipid ROS accumulation (Supplementary Fig. 2E, F). Taken together, sh-PNO1 could partly induce ferroptosis by inhibiting the synthesis of GSH.

### Autophagy induced by PNO1 mediates GSH metabolism reprogramming

**Fig. 3:**
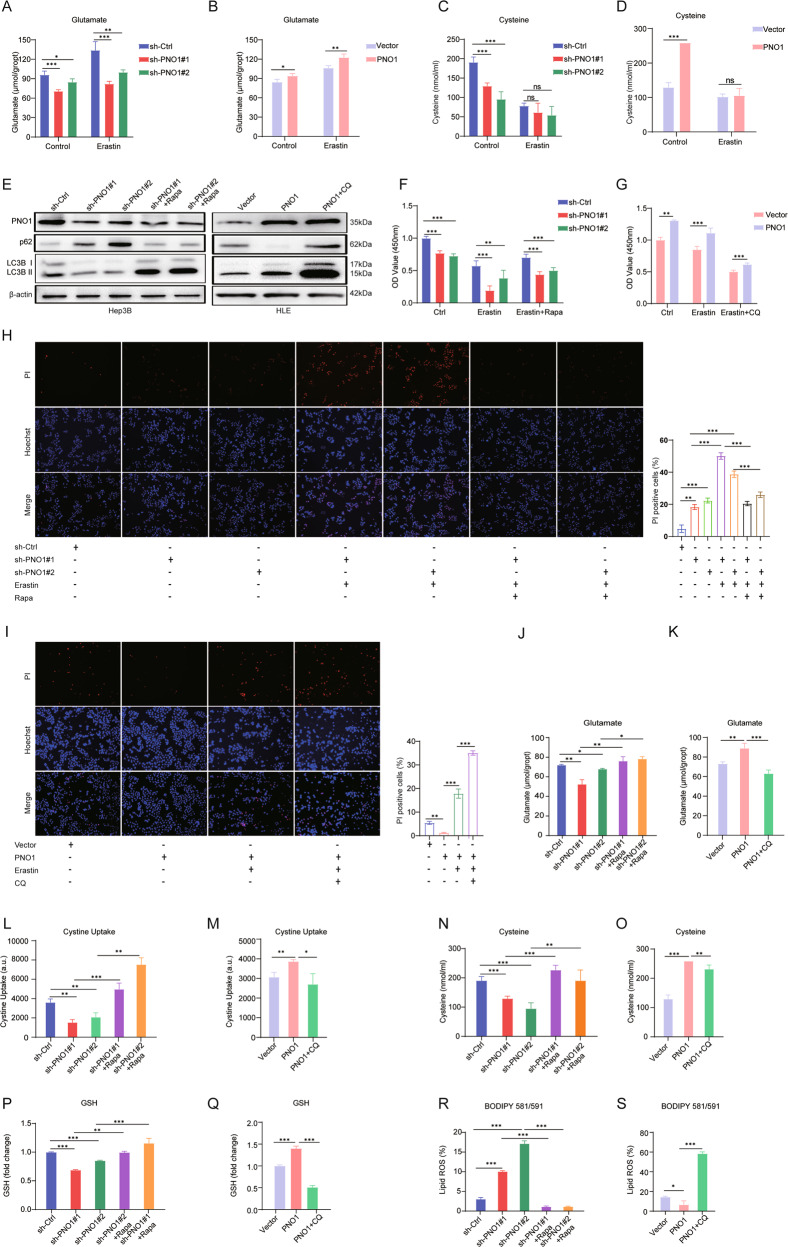
PNO1 regulates the metabolism biosynthesis of GSH through autophagy. **A**, **B** The intracellular glutamate levels of indicated Hep3B (**A**) and HLE (**B**) cells treated with or without erastin (5 μM for Hep3B, 10 μM for HLE) for 24 h (*P < 0.05, ***P < 0.001, **P < 0.01). **C**, **D** The cysteine levels of indicated Hep3B (**C**) and HLE (**D**) cells treated with or without erastin (5 μM for Hep3B, 10 μM for HLE) for 24 h (***P < 0.001). **E** Autophagy-related protein levels in Hep3B and HLE cells by Western Blotting. **F**, **G** Cell viability was assayed in indicated Hep3B (**F**) and HLE (**G**) cells treated with or without erastin (5 μM for Hep3B, 10 μM for HLE) and rapamycin (25 μM for Hep3B) or CQ (25 μM for HLE) for 24 h (***P < 0.001, **P < 0.01). **H**, **I** Cell death was assayed in indicated Hep3B (**H**) and HLE (**I**) cells treated with or without erastin (5 μM for Hep3B, 10 μM for HLE) and rapamycin (25 μM for Hep3B) or CQ (25 μM for HLE) for 24 h (***P < 0.001, **P < 0.01). **J**, **K** The intracellular glutamate levels of indicated Hep3B (**J**) cells treated with or without rapamycin (25 μM) for 24 h and indicated HLE (**K**) cells treated with or without CQ (25 μM) for 24 h (**P < 0.01, *P < 0.05, ***P < 0.001). **L**, **M** The cystine uptake levels of indicated Hep3B (**L**) cells treated with or without rapamycin (25 μM) for 24 h and indicated HLE (**M**) cells treated with or without CQ (25 μM) for 24 h (***P < 0.001, **P < 0.01). **N**, **O** The cysteine levels of indicated Hep3B (**N**) cells treated with or without rapamycin (25 μM) for 24 h and indicated HLE (**O**) cells treated with or without CQ (25 μM) for 24 h (***P < 0.001, **P < 0.01). **P**, **Q** The relative GSH levels of indicated Hep3B (**P**) cells treated with or without rapamycin (25 μM) for 24 h and indicated HLE (**Q**) cells treated with or without CQ (25 μM) for 24 h (***P < 0.001, **P < 0.01). **R**, **S** The lipid ROS levels of indicated Hep3B (**R**) cells treated with or without rapamycin (25 μM) for 24 h and indicated HLE (**S**) cells treated with or without CQ (25 μM) for 24 h (***P < 0.001, *P < 0.05).

GSH is crucial in ferroptosis because it can eliminate the lipid ROS, and GSH is well known to be synthesized by glutamate, cysteine, and glycine [22, 23]. Then, we attempted to address the mechanism underlying GSH synthesis and PNO1-mediated ferroptosis. We found that erastin treatment would upregulate the concentrations of intracellular glutamate (Fig. 3A, B) and downregulate cysteine levels (Fig. 3C, D). Moreover, low expression of PNO1 reversed the upregulation of glutamate concentration induced by erastin treatment but not influencing the concentration of cysteine (Fig. 3A, C). In addition, PNO1 could still affect the intracellular glutamate levels (Fig. 3B) but not the cysteine levels (Fig. 3D) when treated with erastin. On the whole, the above results suggested that PNO1 mainly promotes the levels of intracellular glutamate in HCC cell lines to regulate the synthesis of GSH.

In addition, studies shown that the metabolism of glutamate is related to ferroptosis [24], and high level of intracellular glutamate could enhance system Xc^-^ activity [25]. In our previous study, we investigated that PNO1 promote autophagy in HCC via the MAPK signaling pathway [15]. Mukhopadhyay et al. found that autophagy inhibition diminished the activity of system Xc^-^ in PDAC [26]. Then, we treated HCC cells with the autophagy inducer rapamycin and the autophagy inhibitor chloroquine (CQ) to determine whether PNO1-mediated autophagy is related to the activity of system Xc^-^ in HCC. First, we detected the levels of autophagy-related proteins via Western Blotting. The low expression of PNO1 downregulated the levels of LC3B II and p62, the major indicators in the development of autophagy [27], as expected, the results could be reversed by autophagy inducer rapamycin. PNO1 overexpression increased LC3B II and p62 levels, which could be further increased by CQ (Fig. 3E). Taken together, the results suggested that PNO1 promote autophagy in HCC, consistence with our previous study [15]. To determine whether PNO1-mediated autophagy is related to ferroptotic cell death in HCC cancer cells, we used rapamycin or CQ co-treated with erastin to verify cell viability and cell death through CCK8 (Fig. 3F, G) and PI staining (Fig. 3H, I) experiments. Moreover, the levels of intracellular glutamate and cystine uptake downregulated by sh-PNO1 were reversed after treatment with rapamycin (Fig. 3J, L). In addition, in the parental and HLE PNO1 cells treated with CQ, the high levels of intracellular glutamate and cystine uptake induced by PNO1 could be reversed (Fig. 3K, M). These results verified our hypothesis that PNO1-induced autophagy promotes the concentrations of intracellular glutamate and then activates system Xc^-^. Consistently, the cysteine and GSH levels (Fig. 3N–Q) and lipid ROS levels (Fig. 3R, S) indicated that PNO1-induced autophagy highly activated GSH biosynthesis to inhibit lipid ROS generation. These data collectively suggested that PNO1-induced autophagy inhibits ferroptosis in HCC cells mainly via GSH metabolic reprogramming.

### PNO1 inhibition promotes ferroptosis partly via repressing SLC7A11 expression

**Fig. 4:**
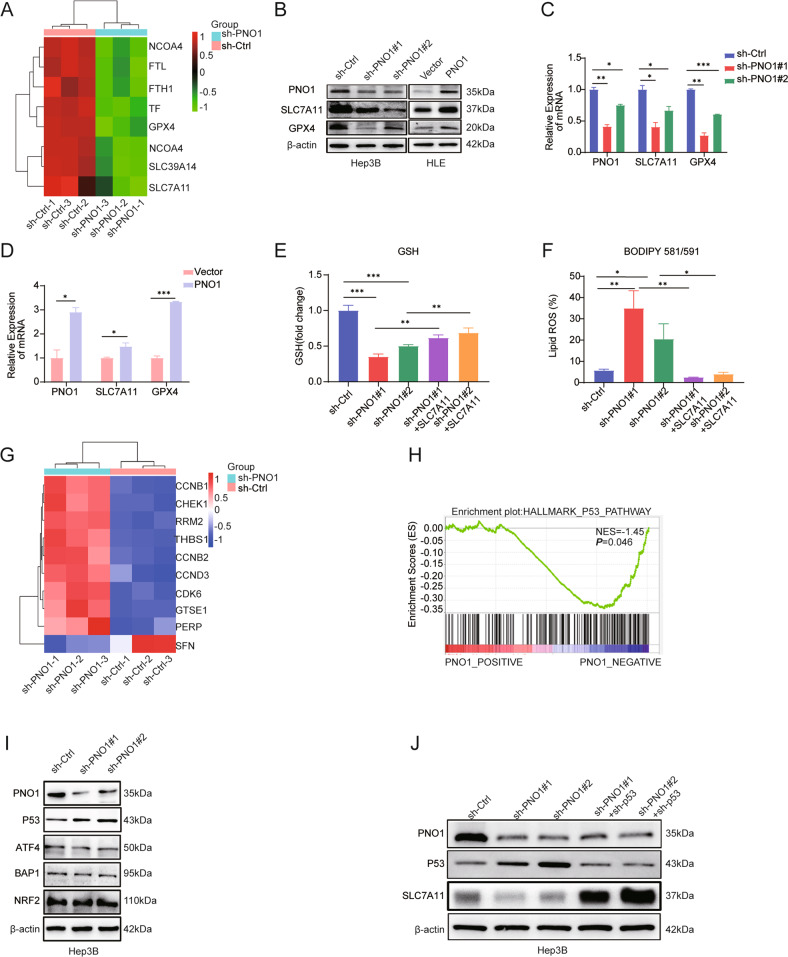
PNO1 regulates the expression of SLC7A11 via p53. **A** Cluster analysis of the correlation between PNO1 expression and ferroptosis-related proteins. **B** Western Blotting analysis of ferroptosis-related protein levels in indicated Hep3B and HLE cells. **C**, **D** The mRNA expression of ferroptosis-related genes was shown in indicated Hep3B (**C**) and HLE (**D**) cells (*P < 0.05, **P < 0.01, ***P < 0.001). **E** The relative GSH levels of Hep3B sh-Ctrl and sh-PNO1 cells treated with SLC7A11 recombinant protein (***P < 0.001, **P < 0.01). **F** The lipid ROS levels of Hep3B sh-Ctrl and sh-PNO1 cells treated with SLC7A11 recombinant protein (*P < 0.05, **P < 0.01). **G** Heatmap showing the correlation between expression of PNO1 and p53 pathway-related proteins. **H** GSEA showing the correlation between PNO1 expression and the p53 pathway genes. **I** The protein levels of SLC7A11 transcriptional regulators in indicated Hep3B cells. **J** The protein levels of SLC7A11 in sh-p53 cells.

Genes linked to the ferroptosis signaling pathway in Hep3B control and sh-PNO1 cells were observed in detail through KEGG enrichment analysis based on our proteomics analysis to further investigate the function of PNO1 in ferroptosis regulation. Notably, SLC7A11 and GPX4 genes associated with PNO1-triggered ferroptosis were mainly enriched (Fig.4A). Considering that SLC7A11 is the catalytic subunit of system Xc^-^, the expression of SLC7A11 is related to the activity of the system to a certain extent [9, 28]. Consistent with mRNA expression levels (Fig.4C and D), SLC7A11 and GPX4 protein levels were markedly reduced by sh-PNO1 and showed the opposite effect under PNO1 overexpression (Fig. 4B). A tissue microarray of a large cohort of 128 HCC samples was evaluated through immunohistochemistry to further explore the expression pattern of SLC7A11 in patients with HCC. As expected, SLC7A11 was positively correlated with PNO1 expression in HCC tumor tissues (*p* < 0.05; Supplementary Fig. 1A, B). Furthermore, SLC7A11 overexpression was associated with poor disease-free survival and overall survival (*p* < 0.001) (Supplementary Fig. 1C, D). Consistently, some studies have shown that the overexpression of SLC7A11 suppresses ferroptosis and that SLC7A11 is highly expressed in human tumors [28]. The SLC7A11 recombinant protein was added to sh-PNO1 Hep3B cells to demonstrate that ferroptosis regulation by PNO1 is specifically dependent on system Xc^-^ (Supplementary Fig. 1E). The addition of SLC7A11 recombinant protein partially restored GSH levels and mitigated lipid peroxidation (Fig. 4E, F). These results suggested that the activity of system Xc^-^ represents an important mechanism in sh-PNO1-induced ferroptosis. Then, to investigate how PNO1 regulated SLC7A11 expression, we explore the KEGG pathway enrichment analysis and found that p53 pathway was the enriched signaling pathways (Fig. 1A). And further analysis revealed that PNO1 was negatively correlated with p53 pathway (Fig. 4G, H). Jiang et al. have demonstrated that p53 is the transcriptional regulator of SLC7A11, the research shown that p53 activation reduced SLC7A11 protein levels [28]. Previous studies also revealed that BAP1, NRF2 or ATF4 are all the transcriptional regulators of SLC7A11 [29–31]. Consequently, we examined protein levels of p53, ATF4, BAP1, and NRF2, the results shown that sh-PNO1 upregulated p53 expression but had no significant effect on the others (Fig. 4I). Then we knockdown p53 expression in sh-PNO1 cell lines, and found the protein level of SLC7A11 was upregulated significantly (Fig. 4J). Taken together, our results suggest that PNO1 regulated SLC7A11 transcription primarily through p53 in HCC.

### The inhibition of PNO1 can synergistically enhance sorafenib-induced ferroptosis

**Fig. 5:**
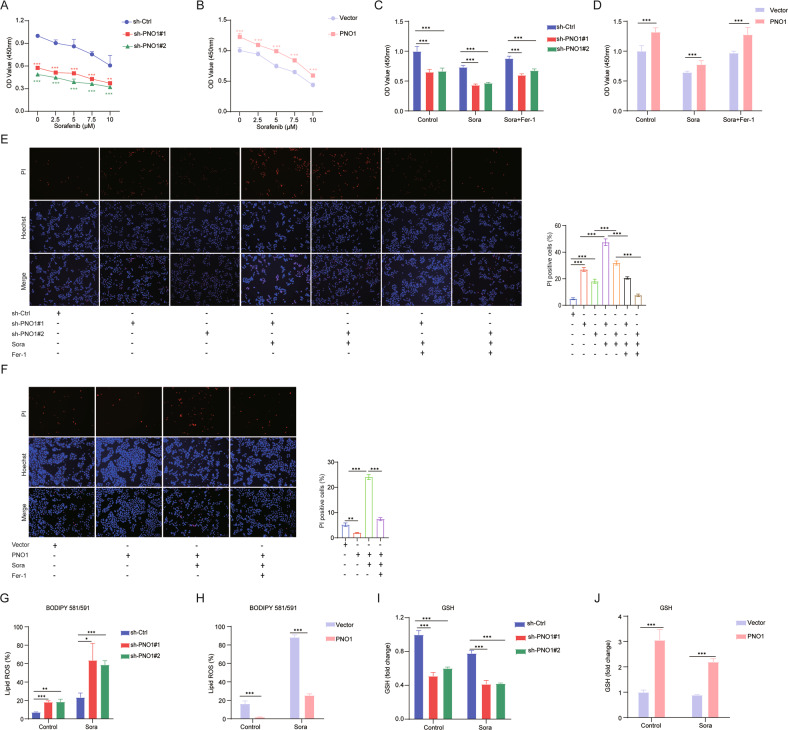
PNO1 inhibition enhances vulnerability of the ferroptosis induced by sorafenib. **A**, **B** Cell viability was assayed in Hep3B sh-PNO1 cells (**A**) and HLE cells (**B**) treated with or without sorafenib (0–10 μM) for 24 h controlled with their parental cells (***P < 0.001). **C**, **D** Cell viability was assayed in indicated Hep3B (**C**) and HLE (**D**) cells treated with or without sorafenib (5 μM for Hep3B, 10 μM for HLE) and ferrostatin-1 (5 μM) for 24 h (***P < 0.001). **E**, **F** Cell death was assayed in indicated Hep3B (**E**) and HLE (**F**) cells treated with or without sorafenib (5 μM for Hep3B, 10 μM for HLE) and ferrostatin-1 (5 μM) for 24 h (***P < 0.001). **G**, **H** The lipid ROS levels were assayed in indicated Hep3B (**G**) and HLE (**H**) cells treated with or without sorafenib (5 μM for Hep3B, 10 μM for HLE) for 24 h (**P < 0.01, ***P < 0.001, *P < 0.05). **I**, **J** The relative GSH levels were measured in indicated Hep3B (**I**) and HLE (**J**) cells treated with or without sorafenib (5 μM for Hep3B, 10 μM for HLE) for 24 h (***P < 0.001).

Sorafenib, an anticancer drug, has been reported to induce ferroptosis via system Xc^-^ in cancer cells [19]. However, sorafenib resistance appears quickly after treatment, so it is badly needed to find a strategy to increase sorafenib efficiency. Thus, we next to investigate if targeting PNO1 can enhance the therapeutic effect of sorafenib. Then, we treated HCC cells with various concentrations of sorafenib to induce ferroptosis. Sorafenib-induced ferroptotic cell death was obviously enhanced by the suppression of PNO1 (Fig. 5A) and weakened by the overexpression of PNO1 (Fig. 5B). Given the above results, we treated Hep3B sh-PNO1 cells with 5 μM sorafenib and HLE PNO1 cells with 10 μM sorafenib for further experiments. Additionally, we treated cancer cells with sorafenib with or without ferrostatin-1 to test whether the increase in ferroptotic cell death was specifically caused by sorafenib. Obviously, the cell viability (Fig. 5C, D) and cell death (Fig. 5E, F) of cancer cells could be reversed after treatment with ferrostatin-1. As shown in Fig. 5G, H, strong evidence was found that PNO1 suppression increases sorafenib-induced lipid ROS accumulation and that PNO1 overexpression decreases lipid ROS levels. Further results suggested that the downregulation of GSH levels induced by sorafenib were enhanced by PNO1 inhibition and weakened by PNO1 overexpression (Fig. 5I, J). The above evidence suggested that treatment with sh-PNO1 could be a potential strategy against sorafenib-induced ferroptosis.

### PNO1 synergizing with sorafenib to induce ferroptosis in vivo

**Fig. 6:**
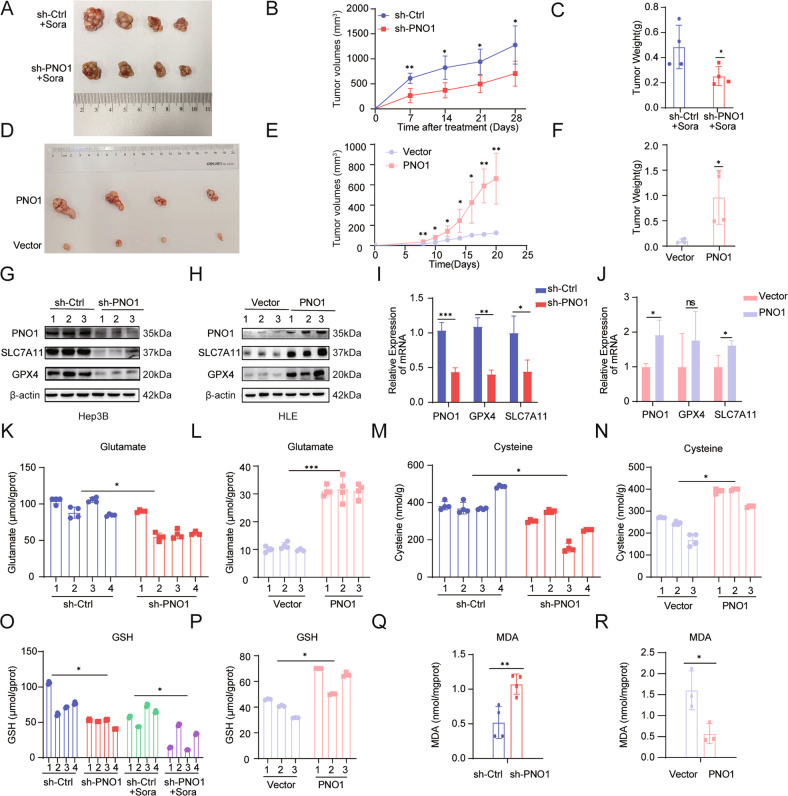
PNO1 promotes ferroptotic cancer cell death in vivo. **A**–**C** Images of tumors from nude mice (**A**), Tumor volumes (**B**), and weights (**C**) in Hep3B sh-ctrl and sh-PNO1 groups after treatment with sorafenib (**P < 0.01, *P < 0.05). **D**–**F** Images of tumors from nude mice (**D**), Tumor volumes (**E**), and weights (**F**) in HLE vector and PNO1 groups (**P < 0.01, *P < 0.05). **G**, **H** Western Blotting analysis of ferroptosis-related proteins of Hep3B sh-ctrl, sh-PNO1 groups (**G**) and HLE vector, PNO1 groups (**H**) in established xenograft model assessed. **I**, **J** The mRNA expression of ferroptosis-related genes in Hep3B sh-ctrl, sh-PNO1 groups (**I**) and HLE vector, PNO1 groups (**J**) was observed (***P < 0.001, **P < 0.01, *P < 0.05). **K**, **L** The intracellular glutamate levels were measured in Hep3B sh-ctrl, sh-PNO1 groups (**K**) and HLE vector, PNO1 groups (**L**) (*P < 0.05, ***P < 0.001). **M**, **N** The cysteine levels were assayed in Hep3B sh-ctrl, sh-PNO1 groups (**M**) and HLE vector, PNO1 groups (**N**) (*P < 0.05). **O**, **P** The relative GSH levels of Hep3B sh-ctrl, sh-PNO1 groups (**O**) and HLE vector, PNO1 groups (**P**) were shown (*P < 0.05). **Q**, **R** The relative MDA levels of Hep3B sh-ctrl, sh-PNO1 groups (**Q**) and HLE vector, PNO1 groups (**R**) were detected (**P < 0.01, *P < 0.05).

Nude mice were subcutaneously injected with Hep3B sh-PNO1 and HLE PNO1 cells and their control cells (5 × 10^6^ cells/mouse) to further validate the oncogenic activity of PNO1 in vivo. Tumor volumes were measured every other day from the second week after the injection. All mice were sacrificed at the end of the fourth week. The primary tumors are shown in Fig. 6A, D. In accordance with our previous research, tumor weights and sizes were higher in the sh-Ctrl group than in the sh-PNO1 group. Notably, the results of tumor weights and sizes provided in Fig. 6B, C suggested that sh-PNO1 significantly enhanced the sorafenib sensitivity of HCC cells. Similarly, high tumor weights and sizes were observed in the HLE PNO1 group (Fig. 6E, F). Moreover, WB analysis showed that the protein levels of the ferroptosis-related indicators SLC7A11 and GPX4 were higher in the Hep3B sh-Ctrl and HLE PNO1 groups than in other groups (Fig. 6G, H). Subsequently, we examined ferroptosis-related indicators through qRT-PCR and obtained similar results (Fig. 6I, J). Compared with the control treatment, sh-PNO1 treatment significantly decreased intracellular glutamate (Fig. 6K) and cysteine (Fig. 6M) levels, whereas PNO1 overexpression increased intracellular glutamate and cysteine levels (Fig. 6L, N). Moreover, GSH levels were obviously decreased in the group under treatment with sh-PNO1 combined with sorafenib (Fig. 6O). In line with the previous results, GSH levels were higher in the HLE PNO1 group than in other groups (Fig. 6P). In addition, the accumulation of malondialdehyde (MDA), a product of lipid peroxidation similar to lipid ROS, increased in the Hep3B sh-PNO1 group and decreased in the HLE PNO1 group (Fig. 6Q, R). In conclusion, PNO1 overexpression promoted HCC proliferation and inhibited ferroptosis, whereas PNO1 inhibition promoted ferroptosis in vivo.

### The working model depicting the role of ferroptosis in PNO1 inhibition

**Fig. 7:**
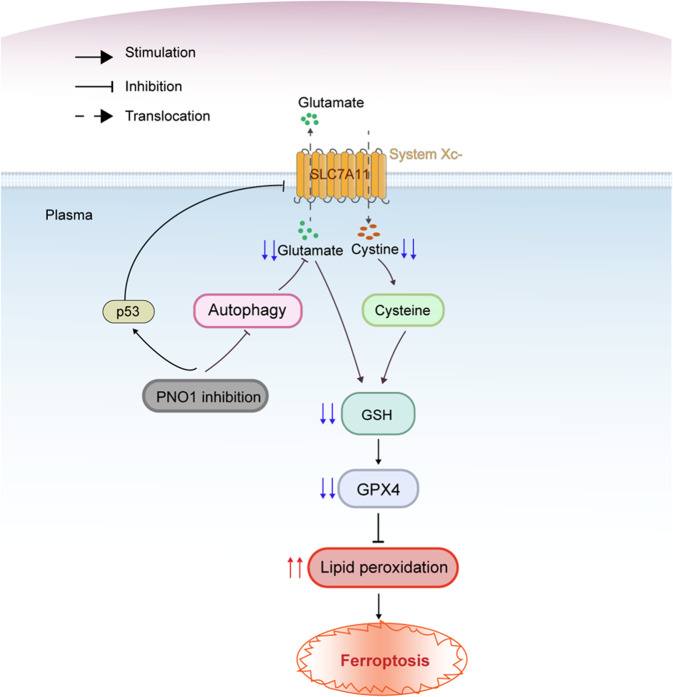
A scheme shown the role of PNO1 on the regulation of ferroptosis in HCC. PNO1 induced autophagy promotes the accumulation of intracellular glutamate, which enhanced the system Xc^-^ activity followed upregulating cysteine level. This leads to GSH metabolism biosynthesis and finally inhibit ferroptosis. PNO1 suppression downregulates the accumulation of GSH and induces ferroptosis in HCC.

We concluded that PNO1 inhibits autophagy-mediated ferroptosis via GSH metabolic reprogramming as demonstrated above. The increased levels of intracellular glutamate generated by PNO1-induced autophagy activate system Xc^-^ and import additional cysteine, finally upregulating GSH biosynthesis against ferroptosis. We also demonstrated that PNO1 inhibition repressed SLC7A11 through p53 to promote ferroptosis. These observations suggested that sh-PNO1 could be a new target in HCC therapy (Fig. 7).

## Discussion

Despite the advances in the research and medical treatment of HCC, therapeutic strategies for this malignancy remain limited. Given the special metabolic functions of liver, the dysregulation of metabolic products could lead to various diseases, including cancer [3]. When tumor cells are exposed to metabolic or oxidative stress, they would trigger the inhibition of ROS, leading to the induction of drug resistance [4]. Therefore, exploring the molecular mechanisms of the redox balance of cancer cells is necessary to overcome the limitations of therapy.

A growing number of studies have attempted to understand the molecular mechanisms of ferroptosis, which may lay the foundation for ferroptosis-targeting therapy. Many studies have already suggested that cellular metabolism is tightly linked to the regulation of ferroptosis and unsurprisingly involves amino acid metabolism [24, 32, 33]. For example, cysteine starvation could induce ferroptosis by downregulating GSH levels because cysteine is the limiting amino acid in GSH synthesis [34]. Additionally, glutamate levels can regulate system Xc^-^ because glutamate is exchanged for cysteine equally through system Xc^-^, the system contactor that regulates ferroptosis. Our study confirmed that PNO1 overexpression leads to the accumulation of intracellular glutamate, which would activate system Xc^-^ to import additional cysteine [25] and eventually increase the biosynthesis of GSH. Finally, the GSH metabolic reprogramming triggered by PNO1 protects HCC cancer cells from ferroptosis. The experiments presented here demonstrated that PNO1 is a negative regulator of ferroptosis in HCC cells.

Moreover, ferroptosis is associated with autophagy [35], which is described as the process of recycling degradation products, maintaining cell homeostasis, and recombining protein and amino acids [36]. Therefore, we posited that cellular metabolism may be the potential bond between ferroptosis and PNO1-induced autophagy in HCC. Most of the current research has revealed that mechanistically, autophagy stimulates ferroptosis mainly via autophagy genes and that other related signaling pathways promote ferroptosis [37, 38]. Although our experimental results have obviously shown that autophagy inhibits ferroptosis, we could not find the mechanistic explanation for this effect on the basis of present research. Notably, Kuanqing Liu et al. revealed that in budding yeast, autophagy promotes glutamate synthesis by providing ammonium. Their result provides important insight into the role of autophagy in diseases, such as cancer [39]. Moreover, similar metabolic reactions have been observed in breast cancer cells, which can recycle ammonium to synthesize glutamate and support nitrogen anabolism [40]. These studies may present some evidence to support the observations on autophagy-mediated ferroptosis in our experiments. In addition, GSH, as an antioxidant reagent, plays an important role in resistance against ferroptosis. Our study revealed that GSH metabolic reprogramming regulated by PNO1-induced autophagy occurs through the promotion of glutamate and cysteine accumulation. We also observed that lipid ROS levels are regulated by rapamycin and CQ, suggesting the significant correlation between autophagy and ferroptosis induced by PNO1. On the basis of the measured cystine uptake levels after treatment with rapamycin or CQ, we concluded that PNO1-induced autophagy could activate system Xc^-^ by increasing the levels of intracellular glutamate [25]. These results revealed that PNO1 promotes autophagy to inhibit ferroptosis via GSH metabolic reprogramming.

Given that ROS levels are still regulated by PNO1 following treatment by the GPX4 inhibitor RSL3, the effect of PNO1 on ferroptosis might not be perfectly ascribable to the SLC7A11-GPX4 axis. In this study, PNO1 is located upstream of GPX4 and thus would not affect ferroptosis under GPX4 blockage. Although the underlying mechanism is still unclear, we posited that PNO1 might also regulate ferroptosis through other mechanisms in HCC cell lines.

Drug resistance to sorafenib, the first-line clinical medicine for advanced HCC, always occurs immediately in most patients [41, 42]. A growing number of studies have revealed that in various cancers, sorafenib induces ferroptosis by inhibiting system Xc^-19^. Therefore, the suppression of ferroptosis might be a new strategy against sorafenib resistance. In this study, we observed the similar functions of sorafenib in HCC cancer cells. Sorafenib treatment substantially increased lipid ROS levels but decreased GSH levels in HCC cells. Additionally, our study found that sh-PNO1 enhances the effect of sorafenib treatment on ferroptosis in vitro and in vivo.

Taken together, our results confirmed that PNO1-induced autophagy inhibits ferroptosis via GSH metabolic reprogramming in vitro and in vivo. We also investigated that PNO1 inhibition promoted ferroptosis via repressing SLC7A11 expression. Out provides a potential therapeutic strategy for liver cancer that combines PNO1 targeting and ferroptosis activators.

## Supplementary information

Reproducibility checklist

relevant supplementary file

Supplementary Figure1

Supplementary Figure2

## Data Availability

All data are fully available without restrictions.

## Source data

Source data

## References

[1] Zong J, Fan Z, Zhang Y (2020). Serum tumor markers for early diagnosis of primary hepatocellular carcinoma. J Hepatocell Carcinoma.

[2] He M, Hu J, Fang T, Tang W, Lv B, Yang B, et al. Protein convertase subtilisin/Kexin type 9 inhibits hepatocellular carcinoma growth by interacting with GSTP1 and suppressing the JNK signaling pathway. Cancer Biol Med. 2021;19:90–103. 10.20892/j.issn.2095-3941.2020.0313 PMC8763006 33893729

[3] Chen J, Li X, Ge C, Min J, Wang F. The multifaceted role of ferroptosis in liver disease. Cell Death Differ. 2022;29:467–80. 10.1038/s41418-022-00941-0 PMC8901678 35075250

[4] Watson J (2013). Oxidants, antioxidants and the current incurability of metastatic cancers. Open Biol.

[5] Sun D, Li H, Cao M, He S, Lei L, Peng J (2020). Cancer burden in China: trends, risk factors and prevention. Cancer Biol Med.

[6] Hambright W, Fonseca R, Chen L, Na R, Ran Q (2017). Ablation of ferroptosis regulator glutathione peroxidase 4 in forebrain neurons promotes cognitive impairment and neurodegeneration. Redox Biol.

[7] Lu B, Chen X, Ying M, He Q, Cao J, Yang B (2017). The role of ferroptosis in cancer development and treatment response. Front Pharmacol.

[8] Wang W, Green M, Choi J, Gijón M, Kennedy P, Johnson J (2019). CD8 T cells regulate tumour ferroptosis during cancer immunotherapy. Nature.

[9] Lang X, Green M, Wang W, Yu J, Choi J, Jiang L (2019). Radiotherapy and immunotherapy promote tumoral lipid oxidation and ferroptosis via synergistic repression of SLC7A11. Cancer Discov.

[10] Roh J, Kim E, Jang H, Park J, Shin D (2016). Induction of ferroptotic cell death for overcoming cisplatin resistance of head and neck cancer. Cancer Lett.

[11] Hangauer M, Viswanathan V, Ryan M, Bole D, Eaton J, Matov A (2017). Drug-tolerant persister cancer cells are vulnerable to GPX4 inhibition. Nature.

[12] Louandre C, Marcq I, Bouhlal H, Lachaier E, Godin C, Saidak Z (2015). The retinoblastoma (Rb) protein regulates ferroptosis induced by sorafenib in human hepatocellular carcinoma cells. Cancer Lett.

[13] Hong T, Lei G, Chen X, Li H, Zhang X, Wu N (2021). PARP inhibition promotes ferroptosis via repressing SLC7A11 and synergizes with ferroptosis inducers in BRCA-proficient ovarian cancer. Redox Biol.

[14] Cheong H, Lu C, Lindsten T, Thompson CB (2012). Therapeutic targets in cancer cell metabolism and autophagy. Nat Biotechnol.

[15] Han Z, Liu D, Chen L, He Y, Tian X, Qi L (2021). PNO1 regulates autophagy and apoptosis of hepatocellular carcinoma via the MAPK signaling pathway. Cell Death Dis.

[16] Liu D, Lin L, Wang Y, Chen L, He Y, Luo Y (2020). PNO1, which is negatively regulated by miR-340-5p, promotes lung adenocarcinoma progression through Notch signaling pathway. Oncogenesis.

[17] Cao J, Chen X, Jiang L, Lu B, Yuan M, Zhu D (2020). DJ-1 suppresses ferroptosis through preserving the activity of S-adenosyl homocysteine hydrolase. Nat Commun.

[18] Dixon SJ, Lemberg KM, Lamprecht MR, Skouta R, Zaitsev EM, Gleason CE (2012). Ferroptosis: an iron-dependent form of nonapoptotic cell death. Cell.

[19] Dixon S, Patel D, Welsch M, Skouta R, Lee E, Hayano M (2014). Pharmacological inhibition of cystine-glutamate exchange induces endoplasmic reticulum stress and ferroptosis. eLife.

[20] Yang W, SriRamaratnam R, Welsch M, Shimada K, Skouta R, Viswanathan V (2014). Regulation of ferroptotic cancer cell death by GPX4. Cell.

[21] Cao J, Poddar A, Magtanong L, Lumb J, Mileur T, Reid M (2019). A genome-wide haploid genetic screen identifies regulators of glutathione abundance and ferroptosis sensitivity. Cell Rep.

[22] Chen Y, Zhu G, Liu Y, Wu Q, Zhang X, Bian Z (2019). O-GlcNAcylated c-Jun antagonizes ferroptosis via inhibiting GSH synthesis in liver cancer. Cell Signal.

[23] Lu SC (2009). Regulation of glutathione synthesis. Mol Asp Med.

[24] Gao M, Monian P, Quadri N, Ramasamy R, Jiang X (2015). Glutaminolysis and transferrin regulate ferroptosis. Mol cell.

[25] Wang K, Zhang Z, Tsai H, Liu Y, Gao J, Wang M (2021). Branched-chain amino acid aminotransferase 2 regulates ferroptotic cell death in cancer cells. Cell Death Differ.

[26] Mukhopadhyay S, Biancur D, Parker S, Yamamoto K, Banh R, Paulo J, et al. Autophagy is required for proper cysteine homeostasis in pancreatic cancer through regulation of SLC7A11. Proc. Natl Acad. Sci. USA. 2021;**118**:e2021475118. 10.1073/pnas.2021475118 PMC8017731 33531365

[27] Yan X, Yang L, Feng G, Yu Z, Xiao M, Cai W (2018). Lup-20(29)-en-3β,28-di-yl-nitrooxy acetate affects MCF-7 proliferation through the crosstalk between apoptosis and autophagy in mitochondria. Cell Death Dis.

[28] Jiang L, Kon N, Li T, Wang SJ, Su T, Hibshoosh H (2015). Ferroptosis as a p53-mediated activity during tumour suppression. Nature.

[29] Koppula P, Zhuang L, Gan B (2021). Cystine transporter SLC7A11/xCT in cancer: ferroptosis, nutrient dependency, and cancer therapy. Protein Cell.

[30] Affar E, Carbone M (2018). BAP1 regulates different mechanisms of cell death. Cell Death Dis.

[31] Rojo de la Vega M, Chapman E, Zhang D (2018). NRF2 and the hallmarks of cancer. Cancer Cell.

[32] Stockwell B, Friedmann Angeli J, Bayir H, Bush A, Conrad M, Dixon S (2017). Ferroptosis: a regulated cell death nexus linking metabolism, redox biology, and disease. Cell.

[33] Hayano M, Yang W, Corn C, Pagano N, Stockwell B (2016). Loss of cysteinyl-tRNA synthetase (CARS) induces the transsulfuration pathway and inhibits ferroptosis induced by cystine deprivation. Cell Death Differ.

[34] Conrad M, Sato H (2012). The oxidative stress-inducible cystine/glutamate antiporter, system x (c) (-): cystine supplier and beyond. Amino Acids.

[35] Liu J, Kuang F, Kroemer G, Klionsky D, Kang R, Tang D (2020). Autophagy-dependent ferroptosis: machinery and regulation. Cell Chem Biol.

[36] Zhou Y, Shen Y, Chen C, Sui X, Yang J, Wang L (2019). The crosstalk between autophagy and ferroptosis: what can we learn to target drug resistance in cancer?. Cancer Biol Med.

[37] Zhou B, Liu J, Kang R, Klionsky D, Kroemer G, Tang D (2020). Ferroptosis is a type of autophagy-dependent cell death. Semin Cancer Biol.

[38] Gao M, Monian P, Pan Q, Zhang W, Xiang J, Jiang X (2016). Ferroptosis is an autophagic cell death process. Cell Res.

[39] Liu K, Sutter B, Tu B (2021). Autophagy sustains glutamate and aspartate synthesis in Saccharomyces cerevisiae during nitrogen starvation. Nat Commun.

[40] Spinelli J, Yoon H, Ringel A, Jeanfavre S, Clish C, Haigis M (2017). Metabolic recycling of ammonia via glutamate dehydrogenase supports breast cancer biomass. Science.

[41] Lachaier E, Louandre C, Godin C, Saidak Z, Baert M, Diouf M (2014). Sorafenib induces ferroptosis in human cancer cell lines originating from different solid tumors. Anticancer Res.

[42] Bruix J, Qin S, Merle P, Granito A, Huang Y, Bodoky G (2017). Regorafenib for patients with hepatocellular carcinoma who progressed on sorafenib treatment (RESORCE): a randomised, double-blind, placebo-controlled, phase 3 trial. Lancet.

