# Supplementary material for: PNO1 inhibits autophagy-mediated ferroptosis by GSH metabolic reprogramming in hepatocellular carcinoma
Source: Cell Death Dis. 2022 Nov 29;13(11):1010. doi: 10.1038/s41419-022-05448-7 (PMC9709074; doi:10.1038/s41419-022-05448-7)
Supplement: Supplementary file 2 — relevant supplementary file [file 41419_2022_5448_MOESM2_ESM.docx]

**Supplementary FigS1. PNO1 regulates the expression of SLC7A11.**

**A-B.** IHC analysis of the relationship of PNO1 and SLC7A11 expression in 128 pairs tissue microarrays of HCC specimens (A). Representative images were taken at a magnification of 50 or 25 μM, spearman=0.207, P=0.019 (B).

**C-D.** The analysis of disease-free survival (C) and overall survival (D) for 128 HCC patients were shown.

**E.** Western Blotting analysis of ferroptosis-related proteins in Hep3B sh-Ctrl and sh-PNO1 cells treated with SLC7A11 recombinant protein.

**Supplementary FigS2. PNO1 suppression enhances the RSL3-induced ferroptosis.**

**A-B.** Cell viability was assayed in Hep3B sh-PNO1 cells (A) and HLE cells (B) treated with or without RSL3 (0-10 μM) for 24 h controlled with their parental cells (***P < 0.001, *P < 0.05, **P < 0.01).

**C-D.** Cell viability was assayed in indicated Hep3B (C) and HLE (D) cells treated with or without RSL3 (2.5 μM) and ferrostatin-1 (5 μM) for 24 h (***P < 0.001).

**F-F.** The lipid ROS levels were assayed in indicated Hep3B (E) and HLE (F) cells treated with or without RSL3 (2.5 μM) for 24 h (***P < 0.001, **P < 0.01).
