# Supplementary figures and images for: PNO1 inhibits autophagy-mediated ferroptosis by GSH metabolic reprogramming in hepatocellular carcinoma
Source: Cell Death Dis. 2022 Nov 29;13(11):1010. doi: 10.1038/s41419-022-05448-7 (PMC9709074; doi:10.1038/s41419-022-05448-7)

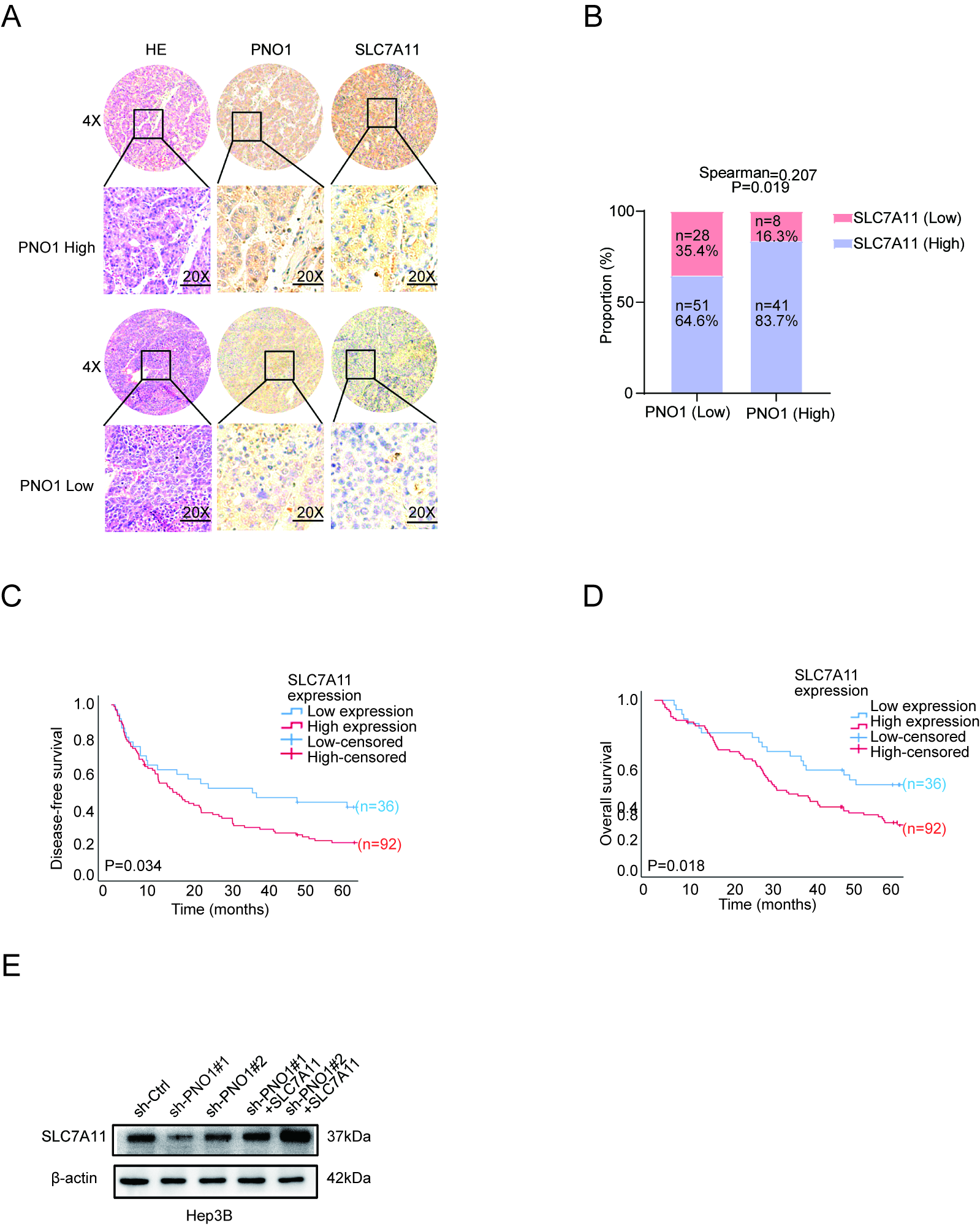

Supplement: Supplementary file 3 — Supplementary Figure1 [file 41419_2022_5448_MOESM3_ESM.tif]

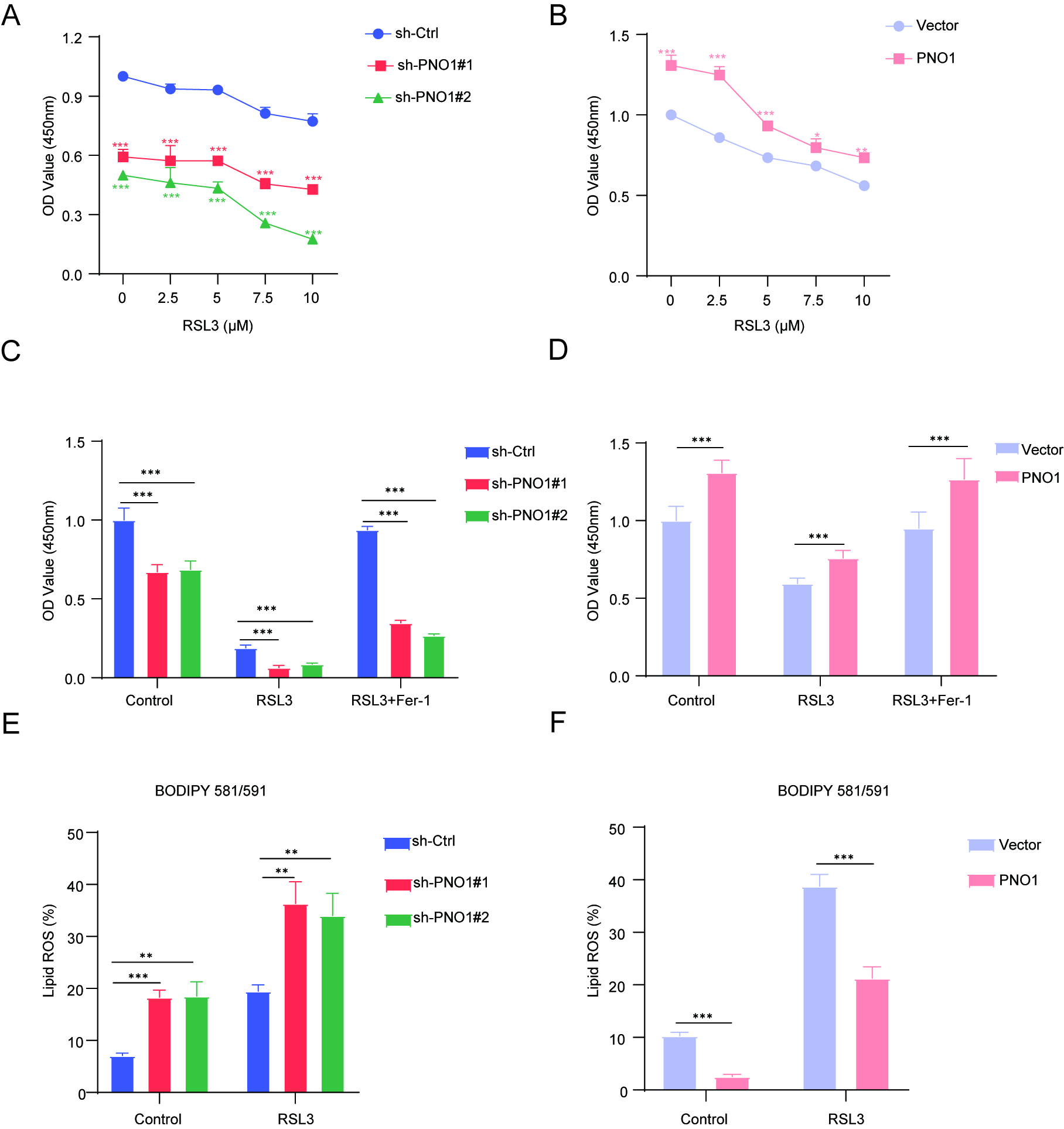

Supplement: Supplementary file 4 — Supplementary Figure2 [file 41419_2022_5448_MOESM4_ESM.tif]
